# “Friction by Definition”: Conflict at Patient Handover Between Emergency and Internal Medicine Physicians at an Academic Medical Center

**DOI:** 10.5811/westjem.2021.7.52762

**Published:** 2021-11-05

**Authors:** Zahir Kanjee, Christine P. Beltran, C. Christopher Smith, Jason Lewis, Matthew M. Hall, Carrie D. Tibbles, Amy M. Sullivan

**Affiliations:** * Beth Israel Deaconess Medical Center, Hospital Medicine Program, Boston, Massachusetts; † Harvard Medical School, Boston, Massachusetts; ‡ Beth Israel Deaconess Medical Center, Carl J. Shapiro Institute for Education and Research, Boston, Massachusetts; § Beth Israel Deaconess Medical Center, Internal Medicine Residency Program, Boston, Massachusetts; ¶ Beth Israel Deaconess Medical Center, Department of Emergency Medicine, Boston, Massachusetts; || Providence Regional Medical Center, Department of Emergency Medicine, Everett, Washington; # Washington State University, Pullman, Washington; ** Beth Israel Deaconess Medical Center, Emergency Medicine Residency Program, Boston, Massachusetts; †† Beth Israel Deaconess Medical Center, Office of Graduate Medical Education, Boston, Massachusetts

## Abstract

**Introduction:**

Patient handoffs from emergency physicians (EP) to internal medicine (IM) physicians may be complicated by conflict with the potential for adverse outcomes. The objective of this study was to identify the specific types of, and contributors to, conflict between EPs and IM physicians in this context.

**Methods:**

We performed a qualitative focus group study using a constructivist grounded theory approach involving emergency medicine (EM) and IM residents and faculty at a large academic medical center. Focus groups assessed perspectives and experiences of EP/IM physician interactions related to patient handoffs. We interpreted data with the matrix analytic method.

**Results:**

From May to December 2019, 24 residents (IM = 11, EM = 13) and 11 faculty (IM = 6, EM = 5) from the two departments participated in eight focus groups and two interviews. Two key themes emerged: 1) disagreements about disposition (ie, whether a patient needed to be admitted, should go to an intensive care unit, or required additional testing before transfer to the floor); and 2) contextual factors (ie, the request to discuss an admission being a primer for conflict; lack of knowledge of the other person and their workflow; high clinical workload and volume; and different interdepartmental perspectives on the benefits of a rapid emergency department workflow).

**Conclusions:**

Causes of conflict at patient handover between EPs and IM physicians are related primarily to disposition concerns and contextual factors. Using theoretical models of task, process, and relationship conflict, we suggest recommendations to improve the EM/IM interaction to potentially reduce conflict and advance patient care.

## INTRODUCTION

### Background

Interactions between emergency physicians (EP) and internal medicine (IM) physicians are frequent and complex. The US Centers for Disease Control and Prevention reported 139 million visits to emergency departments (ED) in the US in 2017; 14.5 million of these resulted in admission, the majority of which were to medical services.^1^ Conflicts in priorities, opinions, and perspectives between these two departments are to be expected.^2^–^4^ The EP makes rapid diagnostic, management, and disposition decisions while simultaneously triaging a high volume of acutely ill pattients; IM physicians, on the other hand, must attend to more detailed workup, diagnosis, and treatment plans while managing bed and staffing resources on the hospital ward.^5^,^6^

Evidence across healthcare settings suggests that suboptimal interdepartmental interactions and inadequately managed conflicts can lead to adverse impacts on patient safety, healthcare systems workflow, physician wellbeing, and employee retention.^7^–^11^ For emergency medicine (EM)/IM interactions specifically, unresolved conflicts and communication failures during patient handoffs between physicians are associated with higher risks of medical errors and adverse events.^9^,^12^–^14^ Understanding the nature of interactions between these groups and optimizing collaboration during patient admission is therefore a high priority for research and, ultimately, patient safety and care.^6^

Although the presence and nature of workplace conflict has been studied in various healthcare settings, rigorous research specifically aimed at elucidating the nature of conflict in the EM/IM interaction remains limited.^15^,^16^ Expert opinion and consensus have highlighted differences between departments in terms of work demands and culture, such as different levels of attention to detail and comfort with initial clinical ambiguity.^6^,^17^ Others have pointed out the pernicious impacts of a silo mentality between these two groups.^18^ A small survey of Australian EM and IM residents^19^ and an interview study at a US hospital^16^ each found that departments differed in their assessment of the adequacy of patient workup in the ED prior to admission, with IM physicians frequently desiring information beyond that which EPs normally provide.

A survey study at a US academic medical center^5^ also found admitting medical services felt they received inadequate information from EPs, and that EPs frequently felt defensive in their interactions with their admitting medical colleagues. Focus group studies of Canadian EPs and IM and general surgery physicians have shown that familiarity and trust were important determinants of quality of communication between these departments,^20^ and that historical factors, attitudes and values, actions, external stressors, and trust could either produce or mitigate interdepartmental conflict.^3^

### Goals of This Investigation

We aimed to describe and explain the interactions and reasons for conflict between these two groups in the context of EM/IM handoffs. Our goal was to provide empirical evidence to inform interventions to enhance interdepartmental interactions and, ultimately, improve patient and physician outcomes.

**Population Health Research Capsule**

What do we already know about this issue?

*Emergency physicians (EP) and internal medicine (IM) physicians interact frequently. Suboptimal interdepartmental interactions can harm patients and physicians.*

What was the research question?

*What are major reasons for conflict between EPs and IM physicians at patient handoff?*

What was the major finding of the study?

*Contextual factors (eg, limited knowledge of the other/their workflow) and disposition disagreements (ie, if/when/where patients should be admitted) led to conflict.*

How does this improve population health?

*Understanding EP/IM physician conflict informs efforts to improve interactions, potentially enhancing outcomes for both patients and physicians.*

## METHODS

### Study Design

We used constructivist grounded theory,^21^ a primarily inductive approach to understand and describe social processes through systematic and rigorous analysis of participant interviews or focus groups. Because we wanted to understand shared perspectives and experiences among our study participants, we conducted focus groups to explore how EPs and IM physicians experienced the handoff process. We drew on the Consolidated Criteria for Reporting Qualitative Research^22^ to guide our analysis and reporting of findings.

### Study Setting

This study was conducted at Beth Israel Deaconess Medical Center, a large, urban, tertiary care, academic medical center affiliated with Harvard Medical School. The hospital is a Level I trauma center with approximately 700 beds and 40,000 annual discharges. Each year the ED sees over 50,000 patient visits, resulting in over 19,000 admissions, approximately 80% of which are to IM general or subspecialty services. Patients are primarily seen in the ED by EM residents supervised by EM attendings who ultimately make disposition decisions. The IM services are staffed either by residents supervised by hospitalists/other medical subspecialists or by hospitalists without residents. The IM residents rotate in the ED, while EM and IM residents rotate together in the medical intensive care units (ICU) at both the academic hospital and a local community hospital. Otherwise, residents in the two departments do not routinely work side by side.

**Figure 1:**
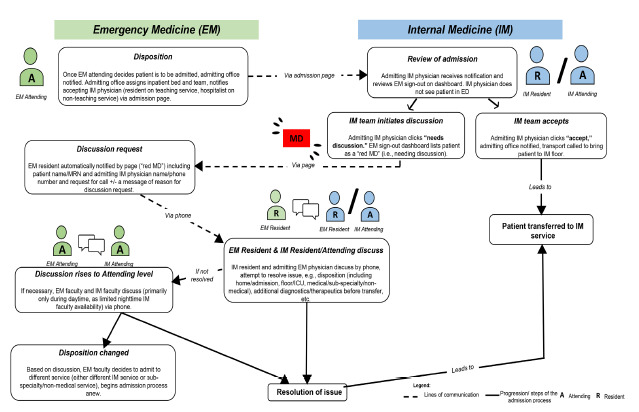
Patient admission process from ED to IM floor. This hospital has designed an electronic signout communication process (e-signout) between EM physicians and admitting physicians through an electronic ED dashboard system. EM attendings decide on need for admission, notify admitting office. Admitting office assigns inpatient bed/team, notifies accepting IM physician via admission page. IM physician (either resident on teaching service, or hospitalist attending on non-teaching service) reviews e-signout and either accepts admission (86% of cases, in which case patient is transferred to IM service) or initiates discussion (14% of cases, in which case a “red MD” notification appears on dashboard and EM resident is notified of need for discussion). If “red MD” case, EM resident and IM resident/attending discuss concerns. If issue is resolved, patient is transferred to IM service. If not resolved, discussion rises to EM attending/IM attending telephone discussion (rare). Issue is either resolved and patient is transferred to IM service or disposition is changed. *EM*, emergency medicine; *IM*, internal medicine; *MRN*, medical record number; *ED*, emergency department.

This hospital has designed an electronic signout communication process (e-signout) between EPs and admitting physicians through an electronic ED dashboard system.^23^ (See Figure 1 for details on the process for admissions to the IM service.) By design, IM physicians rely primarily on the e-signout and do not routinely see admitted patients in the ED, instead meeting and examining them upon arrival to the ward. Both EPs and IM physicians at this facility have reported greatly preferring this system to its verbal handoff predecessor.^24^ The system was designed with a mechanism for inpatient teams to request verbal clarification on signouts whereby a red box with the letters “MD” (known to EM staff as a “Red MD”) appears on the dashboard; in this case, EPs subsequently contact IM physicians for telephone discussion.

Over time, this system has also become a way for inpatient teams to express concerns or requests to EPs. This dashboard is viewable by IM physicians and, for non-ICU admissions, is the only routine admission-related contact between physicians in the two departments. A Red MD discussion request occurred in 14.4% of inpatient medicine admissions during the previous year. In this study, having the dashboard system as the main source of communication between these two departments offered a methodological opportunity, as it provides a unique lens into identifying and understanding sources of conflict between EPs and IM physicians.

### Participant Selection and Data Collection

We conducted focus groups to assess various aspects of the EP/IM physician relationship, including venues for interactions, the nature of the interactions, and suggestions for improvement. Focus group guides (Appendix A) were based on a review of the relevant literature and informed by cognitive interviews^25^ with several faculty experienced in interdisciplinary research at our hospital. Focus group respondents were recruited using purposive sampling via email invitations from department heads and residency program directors and through invitation during departmental faculty meetings. Focus groups were held at times convenient for the participants. Participation was voluntary. Focus groups were conducted until data saturation was reached.

To maximize participants’ sense of psychological safety in the focus groups and interviews, these sessions were conducted in a medical education research space away from clinical areas by two social scientists experienced in qualitative methods (AS and CB). Each focus group was department-specific (EM or IM) and consisted of physicians of the same level (resident or attending). Given the sensitive nature of the topic, we also informed participants that all focus groups were confidential and that the aim of the study was not to place blame on either department, but rather to understand and identify areas for improvement in EM/IM interactions. Sessions were recorded, with recordings sent to a secure human transcription service for deidentified transcription. All transcripts were subsequently reviewed with audio recordings by CB to ensure accuracy. In rare cases, a clinical author (ZK) corrected clinical terminology in the transcripts without listening to the audio recordings to maintain respondent anonymity. Additional observations about non-verbal cues or context were noted by either the focus group facilitator and/or a note-taker, if present, during each session.

### Data Analysis

We analyzed focus group and interview transcripts using the framework approach,^26^ which begins with ongoing inductive content analysis to identify salient themes, followed by organizing themes into matrices.^27^ Matrix displays assist with analysis by visually mapping relationships between participant groups (horizontal axis) and thematic categories identified through content analysis (vertical axis). Specifically, we sought to identify associations between EM and IM respondents to gain a better understanding of how the two departments perceived common areas of conflict that impact their relationships with the other.

Core analytic authors (ZK, AMS, CB) independently read through the transcripts and had ongoing meetings to discuss and identify important themes. Having a core interdisciplinary analytic team composed of a hospital medicine physician (ZK) and social science researchers (AMS, CB) helped to ensure data were interpreted fully and from multiple perspectives. The core analytic team wrote and discussed analytic memos and/or detailed notes for each transcript to document personal reactions (“reflexivity”), identify potential biases and assumptions, and create an audit trail to track decisions and inferences made with these data. To minimize potential IM bias from the core team, several EM authors (JL, MH) read uncoded transcripts independently, generated potential codes, and participated in the analysis at larger team meetings with the core analytic authors to discuss and refine the codebook and data summaries, evaluate the credibility of results, and assess congruence with lived reality of the EM/IM relationship.

After manually marking all transcripts and creating a codebook in Excel (Microsoft Corporation, Redmond, WA), core analytic authors created a matrix display in Word (Microsoft Corporation, Redmond, WA) for each transcript to reduce data into more manageable formats. We further reduced the data by listing areas/sources of interdepartmental conflict along the vertical axis, with participant and topic categories along the horizontal axes as follows: “IM physicians’ perspective of conflict area,” “Emergency physicians’ perspective of conflict area,” “IM physicians’ perspective of emergency physicians,” “Emergency physicians’ perspective of IM physicians,” and “Suggestions for improvement.” Important quotes and synthesized information were entered into overlapping or incongruent perspectives, as well as suggestions discussed by respondents to address each source of conflict.

We used multiple strategies to address trustworthiness or qualitative validity (see Appendix B). Summary reports of the data were shared and discussed with co-authors who held leadership positions in each department (CS and CT). Findings were also presented to EM and IM departmental leaders not involved in the study.

### Ethical Approval

This study received an exempt determination from the Beth Israel Deaconess Medical Center Committee on Clinical Investigations/Institutional Review Board.

## RESULTS

### Characteristics of Study Subjects

See Table 1. From May–December 2019, 24 residents (IM = 11, EM = 13) and 11 faculty members (IM = 6, EM = 5) participated in focus groups. Focus groups with 3–6 participants lasted approximately one hour each (range 33–73 minutes). Due to availability and scheduling needs, two EM faculty members were interviewed one-on-one and one focus group consisted of one physician from each department.

Emergency physicians and IM physicians confirmed that their primary means of interaction was through the e-signout and then, if necessary and requested by the admitting IM physician, subsequently by telephone. Overall, EPs and IM physicians described having effective and collaborative interdepartmental relationships; however, nearly all participants described multiple experiences of preventable conflict and frustration. Although the two departments described different perspectives and expectations of the handoff process, there was considerable agreement within each departmental group about the factors that consistently presented challenges or produced frustration.

### Main Results

We identified two key themes related to the handover interaction: IM physician concerns about patient disposition (Table 2), ie, whether a patient needed to be admitted to the hospital, should go to an ICU, or required additional testing before transfer to the floor) and contextual factors (Table 3) at the level of the individual (the Red MD notification as a primer for conflict, knowledge of the other person and their workflow) and system (clinical workload and volume, the rapid workflow in the ED).

We include representative comments as well as recommendations made by respondents.

### Patient Disposition

#### Whether a patient requires admission at all

Many IM physicians highlighted that they placed discussion requests when they felt that a patient may not need admission. Several agreed that such short-stay admissions necessitated a great deal of effort for limited medical benefit to the patient. One EP said they were “sympathetic” to IM physicians’ concerns and acknowledged that this would be “very frustrating” when IM physicians had “done a whole lot of work to admit a patient who no longer needs admission” (EM attending #2, Focus group F). On the other hand, EPs felt there were often other indications for admission beyond strictly medical reasons that might not be recognized by the accepting IM physician, such as the need for intense education for outpatient management, some of which EPs felt inadequately trained to do. Alternatively, EPs sometimes requested admission to the hospital because an otherwise clinically stable patient was not currently safe to go home.

Sometimes EPs felt that finding “a label to attach” (ie, a diagnosis) to a patient, even if equivocal, made such requests easier. The IM physicians understood the occurrence of label attachment but wished the uncertainty of the label would be more clearly conveyed in the patient sign-out. Many IM physicians wished EPs could more regularly revisit admission decisions made earlier, especially if a patient improved significantly during a prolonged wait for an inpatient bed. Some IM physicians described a perception of futility in discussions to prevent what they thought were unnecessary admissions, which one resident characterized as a “big area of contention” (IM resident #3, Focus group C). However, IM physicians recognized that, due to high patient volume, EPs may not have the time to constantly re-evaluate the need for admission after a patient improves. Several EPs highlighted that prolonged ED patient boarding, and the resultant requirement to cover many patients whom they had not seen, made it especially challenging to overturn a previous EP’s admission request.

#### Whether a patient should go to the ICU rather than the IM service

Several IM physician respondents reported concerns when they felt patients who were admitted to the IM floor would be better served in the ICU. The IM physicians felt that their input on these questions was “undervalued” when they had more firsthand experience than EPs regarding the capabilities and limitations of care on the floor (IM resident #8, Focus group A). On the other hand, EPs felt frustrated that IM physicians were making requests for re-triage without having seen the patient.

#### Whether additional testing is necessary before transfer to floor

Many EPs were frustrated about requests from IM teams for additional testing before patient disposition to the floor. The EPs felt that some of these requests were reasonable (eg, if testing did not require the patient to remain in the ED while awaiting the result or if the doctor was not known to regularly request discussion), while others were perceived as less reasonable (eg, if testing was not going to change acute management or initial disposition) and caused unnecessary patient transfer delays or required significant human resources. One EP explained that some of the conflict around this point was due to “different perceptions of time” (EM attending #2, Focus group F) in the ED vs on the medicine floor, arising from differences in duration of visits in the ED and IM floors. Some EPs felt that requests for additional testing, especially when requested near the end of an IM physician’s shift, were a way to avoid work and pass it on to an oncoming physician. The IM physicians acknowledged these occurrences do happen, though only rarely, and that EPs’ assumptions of these IM physicians being lazy were unjustified.

The IM physicians felt requests for additional testing were important and beneficial to patients. The IM physicians reported asking for more testing because there were resources in the ED to do this more rapidly, rather than having to wait a considerable time for these tests to be done on the ward.

### Contextual Issues

Respondents also identified contextual aspects that contributed to or exacerbated conflict. These occurred on the interpersonal level, with different individual responses to the Red MD dashboard signal and gaps in understanding of the other department physicians’ perspective; and at the hospital systems level, where factors included the impact of high patient volume and the rapid workflow in the ED.

#### Discussion request primes for conflict

The request for additional discussion was often perceived by both EPs and IM physicians as a trigger for conflict because it was used almost exclusively in the context of discussing problems. Internal medicine attendings felt that the interactions were likely perceived as an “implied criticism of [the EP’s] workup” which led to “a defensiveness, which is understandable” (IM attending #3, Focus group B). The EPs expressed similar sentiments and felt especially defensive when such notifications were accompanied by incomplete information in the page about the issue they were being called upon to discuss.

#### Limited knowledge of the other person or their workflow

The EPs and IM physicians expressed that learning about each other personally and their respective workflows could reduce conflict. Direct experience in the ED helped IM physicians appreciate EP perspectives, and social relationships were beneficial to decrease inter-departmental animosity.

#### High clinical workload/volume

Both EP and IM physician respondents cited high patient volume as a significant, or even predominant, stressor on the interdepartmental relationship and physician well-being. High patient volume made what might otherwise be reasonable requests or interactions from their counterparts especially challenging. Citing the heavy workload on both departments, an EM attending reported that the EM/IM “interface is going to be friction by definition, because every side is going to be looking for room [to offload work] from somewhere” (EM attending #2, Focus group F). Both sides felt they did not have a “release valve” to alleviate excessive clinical work.

#### Differing perceptions of the impact of rapid ED workflow

Some IM physicians felt that EPs’ rapid management and disposition decisions could conflict with patient safety. On the other hand, EPs felt that patient safety was the basis for this prioritization of prompt disposition, as ED disposition delays could adversely impact patient outcomes.

### Respondent Recommendations

Respondents provided several recommendations to improve the EM/IM relationship and handover process (Table 4). These included improvements to the signout process (in both documentation and communication), increased positive interdepartmental feedback, guidelines to assist in disposition decisions, and interdepartmental social events.

## DISCUSSION

We conducted a qualitative focus group study of EP and IM physician descriptions of interactions related to patient handoffs at a large academic medical center. In an overall context of positive interdepartmental relationships, we identified patient disposition as a primary point of conflict, specifically the following: 1) whether patients should be admitted at all; 2) whether patients should be admitted to the ICU rather than the medical service; and 3) whether admission should be made pending additional tests in the ED. Contextual factors contributing to conflict included individual and interpersonal issues (discussion request as priming for conflict and lack of knowledge of the other and their workflow) and hospital level factors (high patient volume, and differing perspectives on the impact of rapid ED workflow). In general, these conflicts were not high in intensity, but they did appear regularly in the data and merit attention from physicians in both departments.

**Figure 2:**
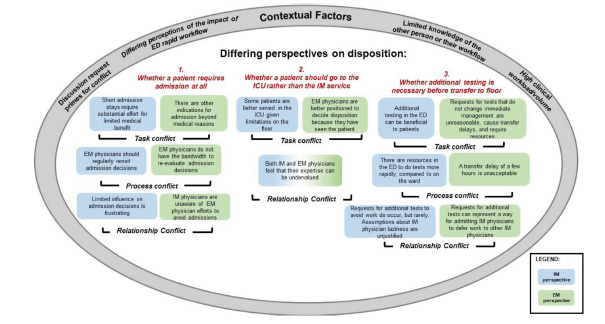
Differences in perspectives regarding disposition decisions result in task, process, relationship conflict between internal medicine physicians and emergency physicians at patient handover. Contextual factors contribute to or exacerbate conflict. *ED*, emergency department; *EM*, emergency medicine; *ICU*, intensive care unit; *IM*, internal medicine

We discuss our findings in the context of current research and theory in organizational and interpersonal conflict, in which conflict has been categorized into subtypes of *task, process*, and *relationship conflict.*^35^,^36^ (See Figure 2 with EP perspectives in green boxes and IM physician perspectives denoted in blue.) Task conflicts include difficulties in achieving mutually satisfactory outcomes because of differences in viewpoints and goals related to the task at hand; these are seen, for example, in conflicts related to IM physician requests to EPs for additional testing (Figure 2, #3). IM physicians described these tests as more efficiently carried out in the ED and ultimately beneficial to the index patient; for EPs, these represented unnecessary transfer delays that would not change patient management and negatively impacted other patients by slowing the ED workflow.

Process conflicts are defined as differing perspectives regarding how tasks should be accomplished. These are exemplified in conflicts arising when IM physicians felt that a particular admission decision should be revisited, whereas EPs felt that their workflow and the safety of other patients would be negatively impacted if they had to continually re-arbitrate initial admission decisions (Figure 2, #1), especially given the detrimental effects of high numbers of ED boarding patients. A third subtype, relationship conflicts, are manifested as tension and frustration between individuals or groups. These can be either antecedent or consequent to task and process conflict. In our study, given the overall positive regard between EPs and IM physicians, relationship conflicts appeared primarily to result from the various task and process conflicts. For example, both IM and EPs felt their expertise was not always being valued when IM physicians raised questions about whether patients should go to the ICU rather than the ward (Figure 2, #2). Understanding the types of conflict present is useful in determining the most appropriate conflict management strategy.

Conflict in the workplace is not uniformly destructive; when managed well, it can also be constructive and enhance productivity and work quality.^37^ A moderate level of task conflict, for example, might improve outcomes by promoting discussion, stimulating critical thinking, and decreasing cognitive biases by incorporating and integrating a diversity of viewpoints. In a recent review of theories of conflict and conflict management,^38^ Tjosvold, Wong, and Chen identified *open-minded discussion* as a foundational contributor to constructive conflict management. They define open-minded discussion as occurring when “people work together to understand each other’s ideas and positions, impartially consider each other’s reasoning for these positions, and seek to integrate their ideas into mutually acceptable solutions.” This aligns with recommendations from our participants to create more opportunities for interdepartmental interactions and discussions, as well as other attempts showing beneficial effects of structured communication between EPs and IM physicians.^39^ The focus, therefore, does not always need to be on eliminating conflict but instead ensuring that all sides can work together productively, respectfully, and efficiently.

Table 4 shows recommendations to reduce negative interprofessional conflict and enhance collaboration between EPs and IM physicians. These recommendations emerge from the respondents themselves (denoted with superscript “a” in the table) and our own inferences and assessments of the key issues. These potential solutions are directed at both individual physicians as well as departments and hospitals and may serve as a starting point for discussion between EPs and IM physicians at other facilities. Several solutions from this list are particularly actionable and generalizable. These include standardizing some disposition decisions via shared interdepartmental working groups who can develop mutually agreeable patient pathways; increasing each department’s understanding of the other and their challenges through interdisciplinary teamwork, conflict training, and social events; and facilitating easier clinical communication in real time, for instance through two-way paging or texting.

## LIMITATIONS

Our study has several limitations. First, this was a single-center study at a large academic medical center, so findings may not be fully transferable to other facilities and settings, although we believe many of the themes we explored are fairly universal. Next, although we made multiple attempts to include attending physicians in our focus groups, our final attending participant counts were relatively low, increasing the possibility that we did not capture the full breadth of views on this topic. Still, each group was represented by multiple physicians and our data reached thematic saturation, suggesting we obtained the key information from our study participants. Support for the validity of our findings is also demonstrated in the multiple and ongoing approaches we employed to maximize trustworthiness of our findings, as detailed in Appendix B. Findings may also be subject to sampling bias because study participation was voluntary and physicians with more or less experience with conflict may have been more likely to participate.

## CONCLUSION

Our focus group study of EP and IM physician interactions related to patient handoff to the medical ward provides a nuanced look at factors related to interdepartmental disagreements and conflicts. Respondents reported largely positive relationships between these groups, yet highlighted conflicts around disposition and contextual factors at both the individual and systems levels which, as one of our participants noted, amounted to “friction by definition.” While our study focused on a single site, the presence of conflict between EPs and IM physicians during patient handoffs is well known outside of our institution, both anecdotally^17^ and in a small number of quantitative studies.^3^,^16^ Our findings extend current research by identifying, in detail, systematic and potentially modifiable causes of conflict and by offering specific suggestions to address these areas of friction. Understanding the perspectives of these two groups of physicians is an important step toward developing effective conflict management strategies and improving collaboration, quality of work life and, ultimately, patient care.

## Supplementary Information



## Figures and Tables

**Table 1:** Characteristics of focus group participants (n = 35).

Characteristic	Total n (% of total)
Department	
Internal Medicine (IM)	17 (48.6)
Emergency Medicine (EM)	18 (51.5)
Respondent group	
IM resident	11 (31.4)
EM resident	13 (37.1)
IM attending	6 (17.1)
EM attending	5 (14.3)
Resident Postgraduate Year (PGY)	
PGY 1	2 (5.71)
PGY 2	10 (28.6)
PGY 3	12 (34.3)
Attending number of years as faculty	
≤ 5 years as faculty	6 (17.1)
> 5 years as faculty	5 (14.3)
Gender	
Male	20 (57.1)
Female	15 (42.9)

**Table 2:** Internal medicine and emergency physician perspectives related to disposition decisions (whether patients require admission at all, whether patients should go to the ICU rather than the IM service, or whether additional testing is necessary before transfer to the floor).*

Topic/perspectives	Representative IM quotes	Representative EM quotes
**Unnecessary admissions** **IM:** It is frustrating to admit people unnecessarily. **EM:** Acknowledgement of this challenge for IM physicians, but emergency physicians do not get credit for the admissions they do prevent.	*Our beds are full and our wards teams are doing their best to discharge everyone that they can safely, but ****when you get a patient who feels fine and wants to go home…it can just get frustrating**** for the patient, for you, for everyone.* IM resident #1, FG C	*We’re not admitting everything. ****We’re trying our best to filter out the [patients who don’t] need to stay**** in the hospital because it doesn’t make sense for [IM physicians] to do work that’s unnecessary.... I think sometimes people forget that.* EM resident #3, FG G
**Attaching a diagnosis to patients admitted for mostly non-medical reasons** **IM:** Non-medical reasons for admission are sometimes appropriate but should be documented. **EM:** Attaching a diagnosis to a patient, even if equivocal, facilitates admission requests.	*...sometimes they’ll put a reason for admission because they’re trying to get the patient upstairs... and the real reason would’ve been more acceptable. They’ll put, “Admit for UTI”...but the ****real reason is the patient...[has] no social supports**** and they’re just not safe to discharge, which is kind of an okay reason to admit somebody…* IM resident #6, FG A	*You’ll often...call something a pneumonia... or call something a UTI that’s kind of borderline. ****If you can find a label to attach, then it’s easier.**** ...If we...could ...just say, “...I really don’t know what’s wrong with this patient, but I don’t think they can safely go home”...that would be much more productive.* EM resident #3, FG D
**Emergency physicians revisiting admission decisions** **IM:** There are instances when revisiting admission decisions would make sense, especially if a patient recovers while waiting in the ED. **Shared:** ED volume and workflow, especially prolonged ED boarding, makes revisiting such admission decisions challenging for emergency physicians.	*There’s a decision...that the patient needs to be admitted...Then the patient sits [in the ED] for 10 hours [during which they become] ****stable and ready to go home****. ….I desperately wish that...the new ...ED team...would be willing to re-evaluate the patient and discharge them...* IM attending #1, FG E	*We’re ****just too busy to re-litigate**** a decision that’s already been made by another resident and attending from our own department.* EM resident #3, FG D *The other issue...is boarding…[Y]our colleague thought [someone] needed to be hospitalized, and you are now the ****3rd or 4th resident...taking care of this patient...waiting for a bed for 18-plus hours.**** Then you get questions from the medicine team about “Do they really need to be hospitalized?”....We’re trying to justify certain things based on how they look now, and that’s just a tough spot to be in*. EM resident #5, FG D
**Perceived futility of IM arguing against need for admission** **Shared:** Emergency physicians rarely reverse admission decisions based on IM physician opinion.	*I don’t actually call anymore if I think the patient should be discharged...****It’s always a lost cause****... they’ve made the decision that the patient needs to be admitted to the hospital and so me...saying, “Have you considered not admitting this patient?” it’s just...a waste of everyone’s time.* IM attending #2, FG B	*I’ve never discharged someone...based on what an internal medicine resident is telling me...they always end up being admitted because ****we have admitting privileges****... At the end of the day, the patient will be coming to them..., which I understand can make them feel [they] have less power….* EM resident #6, FG G
**Personal expertise and perspective regarding ICU disposition** **IM:** IM physicians have knowledge and experience with what is logistically possible on the floor. **EM:** Emergency physicians are the only ones who have seen the patient.	*[O]ur opinion on what [qualifies as] a safe patient for the floor is under-valued. I think ****that’s something that we have more experience than the [physicians in the] emergency room****...We know what it’s like to get a patient from the emergency room on the medicine floor...trying to manage with the limited resources you have, and then trying to transfer that patient [to the ICU].* IM resident #8, FG A	*[Regarding IM teams requesting re-triage to ICU] That can be sort of frustrating because ****that’s coming from somebody who has not seen or evaluated the patient at all**** in person yet, and so we feel like we have the better perspective on that matter.* EM resident #1, FG D
**Effects of transfer delays from IM requests for additional testing** **IM:** Transfer delays are sometimes in the patient’s best interests. **EM:** Transfer delays are experienced differently by emergency and IM physicians.	*They [emergency physicians] thought that [transfer] delay was a bad thing, but...if...we felt...there needed to be a delay, then ****that’s in the patient’s interest.*** IM resident #8, FG A	*There are different perceptions of time...what is a long duration vs a short duration. ****To the emergency department...a [transfer] delay of 2–3 hours is considerable****. It is something we strive to avoid. It’s...not acceptable. A delay of 2–3 hours on the floor isn’t perceptible….[IM physicians say,] “Oh, it’s just a few minutes, just do it.”* EM attending #2, FG F

* **Bolded sections added for emphasis.**

*IM*, internal medicine; *EM*, emergency medicine; *ED*, emergency department; *ICU*, intensive care unit; *FG*, focus group.

**Table 3:** Internal medicine and emergency medicine perspectives on contextual issues that drive interdepartmental conflict.*

Topic/perspectives	Representative IM quotes	Representative EM quotes
**Discussion request as priming for conflict** **Shared:** The discussion request can cause defensiveness, especially for emergency physicians when notifications lack further details.	*That’s the way the system is set up…. The ****discussions are only around conflict**** and never were they, “You did a great job. I’m so impressed with your workup”….It’s only around “Why can’t this patient go home? Are you sure you’ve thought things through?”... Sometimes I just have a small question, but then I’m like, “They’re gonna think that [I have] a criticism, when...I just actually have a question.”* IM attending #2, FG B *It takes a lot of energy on our part to raise that flag because often we know it’s going to be a conflict. You have to feel very strongly...once we already feel strongly, there’s ****already extra emotion**** in there.* IM resident #7, FG A	*That [discussion request] ****relays as contention****...I know me, personally seeing the red MD [icon]...I have a little bit of a block and I go on the defensive....* EM resident #1, FG G *The worst is when they say, “Just please call.”...They don’t give you any information about what their question is...I have no idea what to expect. I’m just going into this conversation blind...Yeah, ****you’re defensive, right off the bat.*** EM resident #4, FG D
**Knowledge of the other person and their workflow** **Shared:** Opportunities to get to know one another personally and their workflows can be helpful.	*[W]e get that the emergency room’s super busy because we...rotate [there]....We know that it’s like a constant flow of patients and that you have five minutes to see a patient, but on the flip side, ****if they rotated with us [on IM services]****, they might see how much pressure there is to discharge patients and the complexities of managing [10–20] sick inpatients at once...* IM residents #2, FG C	*In terms of the actual decreasing animosity during these conversations....it’s, honestly, ****just knowing these people outside of work****. I think that putting a face to a name, having been out to dinner or had a drink with somebody, I think it’s a lot easier to call them.* EM attending # 1, FG H
**Clinical workload/volume** **Shared:** Patient volume makes requests/interactions harder.	*….the issues that we have with the ED stem from that global issue of a ****large number of people trying to be squeezed through**** a tiny little entry point into a thing that has a limited number of beds...Our issues [with emergency physicians] can’t be fixed unless this is fixed…* IM resident #7, FG A *We all think about the pressures on us, but everyone’s pressures...and the volume [keep] going up...****everyone’s already frayed****. Now these innocuous things like, “Hey, can I have more information about the patient?”...are all viewed in the context of, “They’re just making me do more and I don’t have any bandwidth for it.”* IM attending #1, FG B	*[Y]ou’ll get to a point where there’s 25 in the waiting room, 10 in rooms waiting to be seen. At that point you just gotta hustle and get everything done as fast as you can….those are times where we feel the most pressured and those [discussion requests] and stuff ****start to paper cut you a little bit more****.* EM resident #3, FG G
**Release valves** **Shared:** My department does not have a release valve, while my counterparts do.	*It’s not that I’m trying to hold [emergency physicians] to an impossible standard, and not that I’m trying to get out of work. It’s that we’re seeing the other side where there is no release valve. ****Their release valve is us, and our release valve is nothing.*** IM resident #7, FG A	*...If an ambulance is coming [to the ED], you have to make room. You have no ability to turn them away, ask them to go elsewhere. There is ****no release valve.*** EM attending #2, FG F *[In the ED,] it’s not like you can say, “I have to stop working because I have too many patients,” ...that ****generates a lot of friction and animosity**** when you get told [by IM physicians], “Well, I can’t take this patient right now because I’m too busy,” ....because nobody in the emergency department has that option...that generates a lot of friction.* EM attending #1, FG I
**Impact of rapid workflow in ED** **IM:** Emergency physician rapidity can conflict with patient safety. **EM:** Emergency physician rapidity is based on patient safety decisions.	*Their [emergency physicians’] metric is that they’re trying to get people up to the floor as fast as possible... and they don’t always take us seriously when we’re trying to explain the reason why ****we don’t think it’s safe**** for them to go.* IM resident #7, FG A	*I think there’s a perception [of emergency physicians] we’re always into “get ‘em [patients] out [of the ED]”...[I]t’s not appreciated on the medicine side that...a slow [emergency physician] is a dangerous [emergency physician], and that if you let the place get jammed up....then that patient who is in that waiting room with 20 [others] ...actually could be having an acute [myocardial infarction]….That is ****not an economic decision or an efficiency decision. It’s a patient safety decision.*** EM attending #2, FG F

* **Bolded sections added for emphasis.**

*IM*, internal medicine; *EM*, emergency medicine; *ED*, emergency department; *FG*, focus group.

**Table 4:** Problems and recommendations at individual and department/hospital level for reducing emergency/internal medicine physician conflict and enhancing collaboration.

Problem	Individual level recommendation	Department/hospital level recommendation	Comment/rationale
*Problems Related to Disposition*			
Emergency and IM physicians do not have shared understanding of reason for admission (eg, need for intravenous medications, lack of social supports, diagnostic uncertainty), especially when patients were seen by an emergency physician who has since completed their shift (T)	Emergency physicians routinely document specific reason for admission.	Change e-signout template to include specific reason for necessity of disposition decision (rather than alternatives such as home or ICU).	Prevents misunderstandings/disagreements between emergency and IM physicians.
Disposition decisions around need for admission or ICU are sometimes debatable (T)	Emergency and IM physicians work together to create pathways and disposition rules^a^.	Create pathways and disposition rules^a^.	Allows input/expertise of each department in decisions, creates clarity, partially removes these decisions from contentious discussions, capitalizes on complementary inter-departmental knowledge bases.
*Problems Related to Context*			
Disposition discussions approached with defensiveness (R)	Emergency and IM physicians approach each other with curiosity and open-mindedness rather than defensiveness.	Implement interdisciplinary teamwork, conflict negotiation and mitigation training.	Transforms discussion requests from potentially contentious disagreements to satisfying opportunities for interdisciplinary, patient-centered problem solving.
Physicians do not know each other well personally (R)	Emergency and IM physicians attend joint social events^a^ and engage in small talk when able.	Organize joint social events^a^ and trainings.	Facilitates respectful interactions and teamwork.
Physicians do not understand each other’s workflows and priorities well (P)	Emergency and IM physicians ask each other about their priorities and concerns when working together.	Organize joint trainings,^28^,^29^ interdepartmental retreats or workgroups, trainee rotations, and leadership meetings.	Enhances each group’s appreciation of the downstream consequences of their own actions on their counterparts’ lives and work, allowing for emphasis of shared values.
Inpatient demands and inpatient volume make interactions with emergency physicians harder for IM physicians (R)		Reduce strain of admitting and caring for inpatients, eg, through changes to call schedules and geographic admitting, pharmacist involvement in medication reconciliation, streamlined outside record acquisition processes, reduced clinical documentation requirements,^30^ or additional attendings and advanced practice providers.^31^	Reduces strain that challenges IM physicians’ relationships with emergency physicians.
Communication with IM physicians via page/phone is challenging for emergency physicians (P)	IM physicians always provide information on what they need in page for request for more information.	Implement two-way text paging^a^.	Reduces disruption to emergency physician workflow.
Prolonged ED boarding time strains EM/IM interactions (R)		Reduce ED overcrowding and boarding, eg, through strategies such as flexibility in nursing resources,^32^ dedicated hospitalist-led ED boarding teams,^33^ or creation of psychiatry observation units.^34^	Decreases emergency physician stress, makes revisiting admissions decisions easier, reduces likelihood of needing to revisit admission decision made by an off-service emergency physician colleague, and makes discussions with/fulfilling additional requests from IM physicians easier.
Notification of request for information/discussion is perceived as primarily negative by emergency physicians and so is “triggering” (P, R)	IM physicians use request for discussion/information system also to pass on positive feedback^a^.	Adjust e-signout system to include a way to easily provide and encourage positive interdisciplinary feedback.	Makes requests less triggering.

Superscript “a” denotes respondent recommendation.

*IM*, internal medicine; *EM*, emergency medicine; *T*, task conflict; *P*, process conflict; *R*, relationship conflict.
