# Supplementary material for: “Friction by Definition”: Conflict at Patient Handover Between Emergency and Internal Medicine Physicians at an Academic Medical Center
Source: West J Emerg Med. 2021 Nov 5;22(6):1227–39. doi: 10.5811/westjem.2021.7.52762 (PMC8597691; doi:10.5811/westjem.2021.7.52762)
Supplement: Supplementary file 1 [file wjem-22-1227-s001.docx]

**APPENDIX A: FOCUS GROUP GUIDE**

Ground rules: one person at a time, confidentiality, no right/wrong answers, ok to hear positive/negative sides of an issue, men/women equal; any more from group?, Remember that this information is confidential and will not be associated with you individually. Though we want you to be specific, we ask that you do not discuss specific individuals by name.

[3 min] We want to learn more about your experiences interacting with _________ [internal medicine housestaff and attendings when you are in the ED/emergency medicine residents and attendings when you are on the wards]. This does not include interactions that occur during shared rotations when ED residents rotate in the ICU.^^[[1]](#footnote-1)^^ Just to start with some context, l**et’s start with a *brief listing*^^[[2]](#footnote-2)^^ of these types of situations (whether it is in person, electronically, by phone or some other way). We’ll talk later in depth about these types of interactions so for now we can just list the types of situations or circumstances where interactions come up.**

**In which types of situations or circumstances do you interact with housestaff or attendings from ____ [internal medicine/emergency medicine] when you are _____[in the ED/on the wards of the hospital]^^[[3]](#footnote-3)^^?**

[15 min] Recent evidence from the medical school has identified high levels of disrespect across specialties at this hospital.^^[[4]](#footnote-4)^^ I want to ask about disrespect demonstrated between EM physicians and IM physicians and vice-versa. In each of these cases, when I say “physicians” I mean both residents and attendings. Remember that this information is confidential and will not be associated with you individually. Though we want you to be specific, we ask that you do not discuss specific individuals by name.

**How do these data resonate with your experience of the relationship between ED and IM physicians?**

**[Prompt: In what direction is most of the disrespectful treatment?]**

**[Prompt: How often are Internal Medicine physicians treated disrespectfully by Emergency Medicine physicians?]**

1. *What have you experienced personally?^^[[5]](#footnote-5)^^*
   1. *[prompt: without naming names, can you tell me about specific examples?]*
   2. *[prompt: why do you think that other person acted that way?]*
2. *What have you observed personally happening to or by others in your department?*
   1. *[prompt: without naming names, can you tell me about specific examples?]*

**[Prompt: How often are Emergency Medicine physicians treated disrespectfully by Internal Medicine physicians?]**

1. *What have you experienced personally? ^^[[6]](#footnote-6)^^*
   1. *[prompt: without naming names, can you tell me about specific examples?]*
   2. *[prompt: why do you think that other person acted that way?]*
2. *What have you observed personally happening to or by others in your department?*
   1. *[prompt: without naming names, can you tell me about specific examples?]*

- **[Prompt: does this disrespect occur in all of the situations or circumstances during which EM physicians and IM housestaff interact? Or just some of these situations or circumstances? If so, why in those circumstances but not others?]**
- **[Prompt: Is the frequency or nature of disrespect between EM and IM physicians different when there is an imbalance between the level of training of the involved physicians (e.g. an IM attending and ED resident, or an IM resident and ED attending)? How and why?**
- [prompts for moderator on this question if necessary: is experience, perceived competence, confidence, fear of reprisal, fear of getting in trouble, etc involved?]
- [Prompts about types of disrespect for moderator reference only]:
  - Disruptive behavior, humiliating/demeaning treatment; passive-aggressive behavior; passive disrespect^^[[7]](#footnote-7)^^, systemic disrespect^^[[8]](#footnote-8)^^]

[10 minutes] So we have talked about disrespect from internal medicine physicians to emergency medicine physicians, as well as disrespect from emergency medicine physicians to internal medicine physicians.

**What do you think drives these episodes of disrespect between these two groups?**

1. [Prompt if needed: Without placing blame, what part of that is due to the EM physicians?]
2. [Prompt if needed: Without placing blame, what part of that is due to the IM physicians?]
3. [Prompt if needed: What part of this is due to individual persons/personalities versus a larger group dynamic? If persons/personalities, what is it about those specific persons/personalities?]
4. [Prompt if needed: What part of that is due to factors beyond the control of either group?]
5. [Prompt if needed: Where does most of the fault lie?]

- [potential prompts as needed from lit review, primarily for moderator reference]:
  - Patient safety culture
  - Conflicting goals
  - Feeling like other groups are crossing boundaries into the realm of the other group
  - Lack of conflict resolution skills
  - Particular personal conflict resolution management approaches
  - Systemic avenues for conflict management
  - Demographic dissimilarity
  - Lack of trust (eg skills, motives, truth-telling)
  - Poor communication
  - Miscommunication
  - Asymmetric power relations
  - Unclear lines of authority
  - Lack of familiarity
  - personality/charisma, aggressiveness, insecurity
  - Reputation
  - Inappropriate expectations
  - External stressors (eg workload, “production pressure”)
  - Misunderstandings about differences between “positions” and “interests”
  - Ladder of inference

[15 minutes] Now I want to talk about the impacts these interactions (that is, this kind of disrespect between Emergency Medicine physicians and Internal Medicine physicians) have on you, the residents and attendings in your department, and your patients.

**What impacts, if any, do these disrespectful interactions have on your learning? By “your learning” I mean your personal and professional growth in order to be the best doctor you can be.**

- *[prompt: without naming names, can you tell me about specific examples?]*

**What impacts, if any, do these disrespectful interactions have on your quality of life? Prompt: How would you say this affects your risk for burnout?**

- **What impacts, if any, do these disrespectful interactions have on the learning and quality of life of other physicians (residents and attendings) in your department?**
  - [prompt: without naming names, can you tell me about specific examples?]

**What impacts, if any, do these interactions have on patient care?**

- *[prompt: without naming names, can you tell me about specific examples?]*
- *[prompt if necessary: less time for patient care, less motivation for patient care, some other way?*

[10 minutes]^^[[9]](#footnote-9)^^ Often, different medical specialties have reputations within a hospital, sometimes good or bad, sometimes deserved or not. Remember that this information is confidential and will not be associated with you individually. Please do not discuss specific individuals by name.

- [Prompt if needed: reputation can refer to any aspect of how that group is talked about amongst your peers; reputation can include personality, values, competence, etc.]

**In one or two sentences, how would you describe the reputation BI _______[emergency medicine physicians/internal medicine physicians]^^[[10]](#footnote-10)^^ as a whole have amongst people in your department? Why do you think that is?**

**In one or two sentences, what kind of reputation do you think _______ [emergency medicine physicians/internal medicine physicians]^^[[11]](#footnote-11)^^ have amongst ___________[internal medicine physicians/emergency medicine physicians].**

**How do you know? And why do you think that is?**

[10 minutes] We have talked a lot about disrespect today. We would like to talk a bit about respect, as well as potential solutions to this issue.

**Can you think of cases where mutual respect and/or good role modeling has occurred in the case of interactions between EM and IM physicians? Can you provide specific examples? What do you think accounts for these cases?**

**What do you think can be done to improve the relationship between EM physicians and IM physicians?**

**[prompts if necessary: trainings, joint teaching, social outings, changes to electronic systems, increased staffing, conflict training, disciplinary measures, better reporting structures, etc]**

[~5 minutes. Summarize discussion, ask participants to reflect on adequacy of summary.]

**Have we missed anything? Is there anything else that we should have talked about but didn’t?**

Thank you very much for your time.

| **Appendix B.** Strategies used in our study to address qualitative validity, or trustworthiness | | |
| --- | --- | --- |
| **Trustworthiness Criteria** | **Strategies used to address criteria** |  |
| **Credibility**  (Internal validity) | - Focus groups and interviews were conducted until code and thematic saturation were achieved. - Focus groups and interviews were recorded to allow authors to go back to original data if necessary. - Member checks with authors from both the IM and EM department helped to ensure data captured the experiences and perspectives of both departments. - “Thick descriptions” comprised of quotes from the focus group transcripts allows for evaluation of the accuracy of our results. - Quotes or points discussed in earlier focus groups were brought up in later focus groups to evaluate the extent to which respondents agree or disagree with what was discussed by their colleagues. |  |
| **Transferability**  (External validity/generalizability) | - Detailed description of our hospital and dashboard communication system were included to allow readers to evaluate the extent to which our results can be generalized to other contexts. |  |
| **Dependability**  (Reliability) | - Detailed description of our research design and focus group guide were included to allow for replicability. |  |
| **Confirmability**  (Objectivity) | - Maintenance of an audit trail provided a record of the analytic process and decision making that informed our themes and helped to ensure biases did not influence our results. - Team members from each discipline (IM, EM, social science) read uncoded transcripts and generated initial set of codes, created and revised codebook. - Ongoing meetings with a three-person multi-disciplinary team comprised of both a physician (ZK) and non-clinician researchers (AMS & CB) during coding and frequent member checks amongst all members of the research team all throughout helped to ensure findings were interpreted objectively and were free of assumptions. |  |

1. This last sentence should not be read for attendings because attendings don’t rotate in the ICU. [↑](#footnote-ref-1)
2. Emphasis here is on both “brief”, and “listing”. [↑](#footnote-ref-2)
3. NB: We are asking ED doctors about their times interacting with IM doctors when the ED doctors are in the ED. We are asking IM doctors about their times interacting with ED doctors when the IM doctors are on the wards. [↑](#footnote-ref-3)
4. Show graph. [↑](#footnote-ref-4)
5. This a/b and c/d follow up couplets are only asked to a group when you are asking them about their own group receiving disrespect. [↑](#footnote-ref-5)
6. This a/b and c/d follow up couplets are only asked to a group when you are asking them about their own group receiving disrespect. [↑](#footnote-ref-6)
7. Per Leape, this consists of “a range of uncooperative behaviors that are not malevolent or rooted in suppressed anger. Whether because of apathy, burnout, situational frustration, or other reasons, passively disrespectful individuals are chronically late to meetings, respond sluggishly to calls, fail to dictate charts or operating notes in a timely fashion, and do not work collaboratively or cooperatively with others. They resist following safe practices, such as hand disinfection, checklists, and “time-outs,” even when the rationale has been sufficiently described. They may decline to participate in quality improvement efforts, or, if they do, they are indifferent or poor team players. All of these behaviors are manifestations of disrespect—for others, for the institution, and for expert opinions. Although this type of behavior would be included in Hickson’s definition of disruptive behavior,[^14^](https://journals.lww.com/academicmedicine/pages/articleviewer.aspx?year=2012&issue=07000&article=00010&type=Fulltext#R14-10) it is usually not perceived as such by colleagues, who tend to accept it as a fact of life that some people are “difficult.” [↑](#footnote-ref-7)
8. I.e. entrenched systems that show disrespect for a party [↑](#footnote-ref-8)
9. If absolutely necessary, this section can be cut for time. [↑](#footnote-ref-9)
10. Note this is asking about the “other” group within one’s own department, e.g., asking EM physicians about the reputation of IM physicians amongst EM physicians. [↑](#footnote-ref-10)
11. NB this is asking a group to guess what kind of reputation the “other” group has about them, e.g., asking EM physicians what the think IM physicians would say about EM physicians. [↑](#footnote-ref-11)
